# Reaction-Diffusion Modeling ERK- and STAT-Interaction Dynamics

**DOI:** 10.1155/BSB/2006/85759

**Published:** 2006-11-29

**Authors:** Nikola Georgiev, Valko Petrov, Georgi Georgiev

**Affiliations:** 1 Section of Biodynamics and Biorheology, Institute of Mechanics and Biomechanics, Bulgarian Academia of Sciences, Acad. G. Bonchev Street, bl. 4, Sofia 1113, Bulgaria

## Abstract

The modeling of the dynamics of interaction between ERK and STAT signaling pathways in the cell needs to establish the biochemical diagram of the corresponding proteins interactions as well as the corresponding reaction-diffusion scheme. Starting from the verbal description available in the literature of the cross talk between the two pathways, a simple diagram of interaction between ERK and STAT5a proteins is chosen to write corresponding kinetic equations. The dynamics of interaction is modeled in a form of two-dimensional nonlinear dynamical system for ERK—and STAT5a —protein concentrations. Then the spatial modeling of the interaction is accomplished by introducing an appropriate diffusion-reaction scheme. The obtained system of partial differential equations is analyzed and it is argued that the possibility of Turing bifurcation is presented by loss of stability of the homogeneous steady state and forms dissipative structures in the ERK and STAT interaction process. In these terms, a possible scaffolding effect in the protein interaction is related to the process of stabilization and destabilization of the dissipative structures (pattern formation) inherent to the model of ERK and STAT cross talk.

## 

[1,2,3,4,5,6,7,8,9,10,11,12,13,14,15,16,17,18,19,20,21,22,23,24,25,26,27,28,29,30,31,32,33,34,35,36,37,38]
